# Effects of low-frequency repetitive transcranial magnetic stimulation in adductor laryngeal dystonia: a safety, feasibility, and pilot study

**DOI:** 10.1007/s00221-021-06277-4

**Published:** 2021-12-02

**Authors:** Cecília N. Prudente, Mo Chen, Kaila L. Stipancic, Katherine L. Marks, Sharyl Samargia-Grivette, George S. Goding, Jordan R. Green, Teresa J. Kimberley

**Affiliations:** 1 Divisions of Physical Therapy and Rehabilitation Science, Department of Rehabilitation Medicine, School of Medicine, University of Minnesota, Minneapolis, MN USA; 2 Non-Invasive Neuromodulation Laboratory, MnDRIVE Initiative, University of Minnesota, Minneapolis, MN USA; 3 Department of Communication Sciences and Disorders, School of Health and Rehabilitation Sciences, MGH Institute of Health Professions, Boston, MA USA; 4 Present Address: Department of Speech Language Hearing Sciences, Boston University, Boston, MA USA; 5 Department of Communication Sciences and Disorders, University of Minnesota-Duluth, Duluth, MN USA; 6 Department of Otolaryngology-Head and Neck Surgery, University of Minnesota, Minneapolis, MN USA; 7 Present Address: Department of Physical Therapy, School of Health and Rehabilitation Sciences, MGH Institute of Health Professions, 36 First Ave, Boston, MA 02129 USA; 8 Present Address: MicroTransponder Inc., Austin, TX USA; 9 Present Address: Department of Communicative Sciences and Disorders, University at Buffalo, Buffalo, NY USA; 10 Department of Psychiatry and Behavioral Sciences, Medical School, University of Minnesota, Minneapolis, MN USA

**Keywords:** Adductor laryngeal dystonia, Spasmodic dysphonia, Repetitive transcranial magnetic stimulation, Cortical silent period, Acoustics

## Abstract

**Purpose:**

The effects of neuromodulation are virtually unexplored in adductor laryngeal dystonia (AdLD), a disorder characterized by involuntary contraction of intrinsic laryngeal muscles. Recent findings indicated that intracortical inhibition is reduced in people with AdLD. Low-frequency repetitive transcranial magnetic stimulation (rTMS) induces prolonged intracortical inhibition, but the effects in AdLD are unexplored. This pilot and feasibility study aimed to examine the safety, feasibility, and effects of a single session 1 Hz rTMS over the laryngeal motor cortex (LMC) in people with AdLD and healthy individuals.

**Methods:**

The stimulation location was individualized and determined through TMS-evoked responses in the thyroarytenoid muscles using fine-wire electrodes. 1200 pulses of 1 Hz rTMS were delivered to the left LMC in two groups: Control (*n* = 6) and AdLD (*n* = 7). Tolerance, adverse effects, intracortical inhibition, and voice recordings were collected immediately before and after rTMS. Voice quality was assessed with acoustic-based and auditory-perceptual measures.

**Results:**

All participants tolerated the procedures, with no unexpected adverse events or worsening of symptoms. No significant effects on intracortical inhibition were observed. In the AdLD group, there was a large-effect size after rTMS in vocal perturbation measures and a small-effect size in decreased phonatory breaks.

**Conclusions:**

One rTMS session over the LMC is safe and feasible, and demonstrated trends of beneficial effects on voice quality and phonatory function in AdLD. These preliminary findings support further investigation to assess clinical benefits in a future randomized sham-controlled trial.

**ClinicalTrials.gov:**

NCT02957942, registered on November 8, 2016.

**Supplementary Information:**

The online version contains supplementary material available at 10.1007/s00221-021-06277-4.

## Introduction

Adductor laryngeal dystonia (AdLD) (also referred to as spasmodic dysphonia) is the most common form of laryngeal dystonia, a rare disorder characterized by excessive contraction of intrinsic muscles in the larynx. People affected by AdLD often have a strained voice quality and experience phonatory breaks during speech due to intermittent hyper-adduction of the vocal folds (Ludlow et al. 2008). These symptoms impair effective communication, and can have a negative impact on many aspects of patients’ lives, such as occupation and social participation. The most common treatment for AdLD is injection of botulinum toxin into the muscles of the larynx. However, benefits of botulinum toxin injections are only short term and not universally effective (Ludlow et al. 2008). Other treatment options such as voice and speech therapy may be less effective than botulinum toxin injections, and selective laryngeal adductor denervation–reinnervation surgery may be less effective and present greater risk to patients (Ludlow et al. 2008, 2018; Simonyan et al. 2021). A few neuromodulation modalities have been explored, such as deep brain stimulation, vibro-tactile, and electrical laryngeal stimulation (Pitman 2014; Khosravani et al. 2019; Honey et al. 2021). Current priorities indicate the pressing need for novel interventions in laryngeal dystonia (Simonyan et al. 2021).

The cause of AdLD is unknown. Neuroimaging and neurophysiology studies have suggested that AdLD and other focal dystonias are associated with impaired inhibition in sensorimotor cortical areas (Chen et al. 2020; Kimberley et al. 2013, 2015; Lorezon et al. 2016; Pirio-Richardson et al. 2017; Samargia et al. 2014, 2016; Simonyan and Ludlow 2010; Simonyan et al. 2021; Suppa et al. 2015). We developed a method to assess the primary motor cortical representation and excitability for the thyroarytenoid (TA), a laryngeal muscle affected in AdLD, using single-pulse transcranial magnetic stimulation (TMS) delivered to the primary motor cortex and fine-wire electrodes inserted into the TA (Chen et al. 2017). Using this method, we previously compared the cortical silent period (cSP), a TMS-evoked measure associated with intracortical inhibition, in people with AdLD and healthy controls. The findings revealed shorter cSPs in the AdLD group suggesting decreased intracortical inhibition in the laryngeal motor cortex (LMC) (Chen et al. 2020).

In the current study, we sought to conduct a preliminary safety and feasibility investigation on the effects of modulating LMC activity with repetitive TMS (rTMS) in a small group of individuals with AdLD and healthy controls. rTMS at lower frequencies can induce prolonged inhibition in a process analogous to long-term depression (Chen et al. 1997; Gerschlager et al. 2001). Given that AdLD has been found to be associated with decreased cortical inhibition and that 1 Hz rTMS is known to increase intracortical inhibition, the purpose of this pilot study was to examine the effects of 1200 pulses of 1 Hz rTMS delivered to the LMC in people with AdLD and healthy individuals. Safety, feasibility, cortical excitability, and acoustic and auditory-perceptual metrics of voice quality were measured. To our knowledge, this is the first study to examine the effects of rTMS of the LMC in AdLD or healthy controls. We aimed to investigate these methods in a small group of patients with AdLD to examine whether the procedures used for testing (single-pulse TMS with fine-wire placement into the TA) and the intervention (rTMS over the laryngeal motor cortex) were safe and tolerable, and did not exacerbate presenting voice symptoms in a clinical population for whom neuromodulation has been largely unexplored (Simonyan et al. 2021).

We hypothesized that one session of low-frequency rTMS would be tolerable by all participants with no unexpected adverse events, including exacerbation of voice symptoms. We also hypothesized that one session of 1 Hz rTMS would lead to prolongation of the LMC cSP indicating increased inhibition. Given results of our previous work (Chen et al. 2020), we did not anticipate differences in response between the left and right TA. Due to the brief intervention (1 session), we were not examining for clinically significant or lasting changes to voice in either group; however, given this was an exploratory, pilot investigation, even small changes in the direction of improvement offer promising evidence for the continued exploration of such a technique.

## Methods

### Design and blinding

This was an open-label, pilot, single-blinded trial. All study procedures were completed on the same day and in the following order: (1) symptom questions; (2) voice recording; (3) fine-wire electrode insertion; (4) TMS single-pulse pre-testing; (5) rTMS intervention (20 min); (6) TMS single-pulse post-testing; (7) electrode removal; (8) voice recording; (9) symptom questions. Total experiment duration was approximately 90 min. There were no breaks between steps.

Data were de-identified prior to processing and statistical analysis to ensure that all investigators were blinded to group (AdLD vs. Control) and time (pre- vs. post-rTMS).

### Participants

This study was approved by the Institutional Review Board of the University of Minnesota. The study was registered on clinicaltrials.gov (NCT02957942). All participants gave written, informed consent prior to participation according to the Declaration of Helsinki (World Medical Association, 2013).

All participants were screened for any health condition or impairment that would interfere with participation. Subjects were also screened according to TMS safety guidelines (Rossini et al. 2015). People taking selective serotonin reuptake inhibitors or medications that potentially lower seizure threshold were excluded. The breakdown for recruitment and enrollment in both groups is shown in Table 1.

**Table 1:** Recruitment information

	AdLD (*n*)	Control (*n*)
Initial interest in the study	21	23
No response to follow-up contact	0	5
Screened	21	18
Eligible and interested	7	8
Eligible, but unable to schedule visit	4	3
Not eligible	6	5
Not interested	4	2
Enrolled and consented	7	7
Completed experiment procedures	7	6

*AdLD* adductor laryngeal dystonia

Fourteen participants were enrolled. One healthy participant (53 years old, female) was excluded from final analyses due to loss of electromyographic signal during the experiment. The final sample consisted of six healthy controls (53.2 ± 6.6 years; 3 females) and seven participants with AdLD (61.0 ± 3.8 years; 6 females). Eleven participants were right-handed based on subjects’ self-report. Demographic information is listed in Table 2.

**Table 2:** Demographic information

ID	Sex	Age (yrs)	Time since diagnosis (yrs)	Last BTX (days)	Next BTX (days)	VHI Total
Control1	M	51	NA	NA	NA	NA
Control2	F	45	NA	NA	NA	NA
Control3	F	52	NA	NA	NA	NA
Control4	M	62	NA	NA	NA	NA
Control5	M	60	NA	NA	NA	NA
Control6	F	49	NA	NA	NA	NA
AdLD1	F	64	11	77	14.0	61
AdLD2	M	59	5	131	48.0	45
AdLD3	F	65	26	160	8.0	46
AdLD4	F	65	27	48	7.0	74
AdLD5	F	56	28	105	NA	59
AdLD6	F	61	20	58	4.0	61
AdLD7	F	57	1	88	5.0	73

Demographic information for all participants

*AdLD* adductor laryngeal dystonia, *BTX* botulinum toxin injection, *F* female, *M* male, *NA* not applicable, *VHI* Voice Handicap Index,  *yrs* years

Control subjects were screened over the phone for absence of vocal fold pathology based on self-report to exclude potential participants with the following: positive diagnosis for voice disorder, voice tremor, or dystonia; difficulty in speaking, whispering, shouting, laughing, singing, crying, and coughing; loss of voice during a word or sentence; and prior history of laryngeal surgery or chronic gastroesophageal reflux disease. For the AdLD group, participants with a diagnosis of AdLD from a laryngologist and who met criteria regarding their voice deficit were recruited. Diagnostic criteria were based on the recommendations in the Spasmodic Dysphonia Attribute Inventory (SDAI) (Ludlow et al. 2018) and verified by an experienced laryngologist (S. Goding) before each experiment. The average time since diagnosis was 16.9 ± 11.2 years. Participants with AdLD who received regular botulinum toxin injections participated when the benefits of the toxin were at a minimum, per patient report, typically 1–7 days before their regular re-injection schedule. The average time since the last toxin injection was 95.3 ± 39.9 days. Individuals with AdLD combined with voice tremor were excluded due to potential confounding pathology. To categorize severity, self-reported voice disability was assessed at pre-test using the Voice Handicap Index (VHI), which reliably measures the influence of the subjects’ voice disorder on their quality of life across three dimensions (physical, functional, and social) (Jacobson et al. 1997). The mean total VHI score for the AdLD participants was 59.9 ± 11.5, with voice disability classified as either moderate (5/7) or severe (2/7).

### Safety and feasibility

Protocol safety was measured through participant report of adverse events at post-test and 1 day after the rTMS session (i.e., follow-up) via phone call or email. At each time point, participants were asked to report and, if applicable, rate a subset of possible symptoms such as seizures, pain, hearing discomfort, dizziness, and others (Supplement 1). Protocol feasibility was measured by the number of participants able to complete all testing and rTMS procedures, since it was unknown how the prolonged fine-wire placement into the TA muscle would be tolerated by participants.

### Neurophysiology

The neurophysiologic effects of low-frequency rTMS to the LMC were tested in people with AdLD and healthy controls.

#### Cortical silent period (cSP)

Intracortical inhibition was tested at pre- and post-test with the cSP. The cSP is a TMS-based measure of intracortical inhibition thought to originate mainly from the activation of cortical inhibitory interneurons likely mediated by gamma-aminobutyric acid (GABA) receptors (Lorezon et al. 2016; Werhahnetal et al. 1999). The cSP is defined as an interruption of voluntary muscle contraction induced by stimulation of the contralateral motor cortex. The cSP for a given muscle is tested by applying a single suprathreshold TMS pulse to the primary motor cortical representation of the tonically activated muscle, producing a period of silent activity in the target muscle (Wolters et al. 2008). Based on this principle, the duration of the cSP for the bilateral TA muscles was measured during phonation, while TMS was delivered to the left LMC. The left LMC was chosen due to the bilateral cortical control of the TA musculature and known left laterality of most phonation tasks (Simonyan et al. 2012). cSP responses for the TA were collected as previously reported (Chen et al. 2017, 2020). Briefly, after skin preparation, fine-wire electrodes were inserted into bilateral TA muscles via a percutaneous approach. The electrode wires were connected to a bipolar electromyography (EMG) pre-amplifier (Y03, Motion Lab Systems, Inc., LA, USA) with a gain of × 300. The EMG signal was passed through a band-pass filter with cut-off frequencies of 15 Hz and 2000 Hz and then digitized by an analog-to-digital convertor (NI 9234, National Instruments Corporation, Austin, TX, USA) with a resolution of 24-bits at a sampling rate of 6400 Hz. All data were collected and stored using a custom data acquisition program written with LabVIEW (V2012, National Instruments, Austin, TX) on a laptop computer (Latitude, Dell Co., Ltd, Round Rock, TX) which was also used to monitor real-time EMG activity. Single pulse TMS was applied with a 70 mm figure-of-eight remote coil connected to the Magstim BiStim^2^ and 200^2^ stimulator set (Magstim Company LTD, West Wales, UK). The coil was positioned on the participant’s head over the approximate left LMC (Chen et al. 2017, 2020). A brain template was used to assist with coil localization. Localization of the LMC for rTMS delivery was driven by the identification of the TA hotspot defined as the location within the LMC which evoked a visible cSP in 3 out of 5 trials. Cortical stimulation was spatially tracked with neuronavigation (BrainSight, Rogue Research Inc., Quebec, Canada) to decrease variability of stimulus delivery during TMS testing. Stimulation intensity was determined via the cSP threshold, which was defined as the lowest intensity that evoked a visible cSP in 3 out of 5 trials (Chen et al. 2017, 2020). Maximum stimulator output (MSO) for TMS and rTMS cSP thresholds for each participant are listed in Table 3. Thirty trials of cSP were collected from the left hemisphere during phonation of sustained /i/ both at pre- and post-test. Participants were instructed to produce a comfortable pitch and volume that was kept similar throughout the trials.

**Table 3:** Adverse events

A. All participants			
	*N* (% of total events)		
	Post	Follow-up	Total
Throat pain/discomfort	7 (22.6)	3 (9.7)	10 (32.3)
Tenderness on skin (insertion area)	7 (22.6)	6 (19.4)	13 (41.9)
Neck pain	4 (12.9)	1 (3.2)	5 (16.1)
Dental pain/discomfort	1 (3.2)	0	1 (3.2)
Temporary dizziness or fatigue	1 (3.2)	1 (3.2)	2(6.5)
Total	20 (64.5)	11 (35.5)	31 (100)

B. Analysis per group				
	Total events			
	Control		AdLD	
	Post	Follow-up	Post	Follow-up
Throat pain/discomfort	3	2	4	1
Tenderness on skin (insertion area)	4	1	3	5
Neck pain	3	1	1	0
Dental pain/discomfort	0	0	1	0
Temporary dizziness or fatigue	1	1	0	0

Adverse events reported after one session of rTMS

(A) Adverse events reported at post-test (Post) and follow-up by all participants combined. Post-test symptoms were reported at the end of the experiment, while follow-up symptoms were reported 1 day after the experiment via phone or email interview. (B) Adverse events reported in each group at each timepoint

*AdLD* adductor laryngeal dystonia

#### rTMS

rTMS was applied to the left LMC with the AirFilm® (Rapid version) figure-of-eight coil which was connected to the Rapid^2^ Magnetic Stimulator (Magstim Company LTD, West Wales, UK). rTMS delivery was spatially tracked in all participants with neuronavigation (BrainSight, Rogue Research Inc., Quebec, Canada) to decrease variability of stimulus delivery. After the single-pulse TMS pre-testing, the cSP threshold was reassessed with the same process, but with the Rapid^2^ stimulator and the AirFilm® air-cooled coil. This reassessment is necessary due to potential small threshold differences resultant from different coils and is referred to as the ‘rTMS cSP threshold’. Intensity of the rTMS stimulation intervention was set to 90% of the rTMS cSP threshold to ensure sub-motor-threshold stimulation intensities during the repetitive stimulation (Chen et al. 2018), similar to the parameters used by other rTMS studies in hand and masseter muscles (Borich et al. 2009; Huang et al. 2017; Kimberley et al. 2013, 2015). All participants received a single 20-min session of 1 Hz rTMS (1200 pulses, biphasic waveform) following established methods (Borich et al. 2009; Kimberley et al. 2013, 2015). Given that the purpose of this study was to test safety, feasibility, and effect of rTMS, a sham condition was not included.

#### TMS data processing

cSP values for each TA muscle were collected for all participants using TMS single-pulse testing at pre-test and post-test, after the single session of 1 Hz rTMS. Total cSP duration for each TA side was calculated as the time between the onset and offset of the averaged and rectified cSP (Chen et al. 2017, 2020; Wolters et al. 2008) following previously published methods (Chen et al, 2017, 2020). Briefly, the EMG signal collected over the 30 trials of cSP testing for each side was first averaged and rectified. Then, a 10-ms moving standard deviation (SD) window was used to generate an SD curve of the signal. The pre-stimulus (baseline) contraction level was determined by calculating the average value of the SD curve during baseline. The onset of the cSP was defined as the onset of the stimulus, while the offset was defined as the time point when the signal returned to the pre-stimulus level. Three examiners blinded to group assignment and time (pre- vs. post-rTMS) confirmed the cSP offset by visual inspection and the final offset value was determined by consensus between all examiners. Given that EMG recordings were collected for right and left TA muscles before and after rTMS, data processing resulted in a total of four cSP values for each participant (*n* = 52). cSP responses could not be confirmed by the blinded examiners in three cases in the Control group, likely due to poor fine-wire EMG signal. Data for these cases were excluded from the final data analysis and included: left TA pre-test (*n* = 1), left TA post-test (*n* = 1), and right TA post-test (*n* = 1). The final analysis included a total of 49 cSP responses.

### Objective and auditory-perceptual measures of voice

We used well-established metrics of voice quality (both acoustic-based and auditory-perceptual) to test for possible effects of low-frequency rTMS on voice in all participants. Voice recordings were digitally recorded (44.1 kHz sampling rate) before and after rTMS using Audacity® (version 2.1.2) and a headset with a microphone (Plantronics, Blackwire c510-m) and a mouth-to-microphone distance of approximately 1 inch. All voice recordings were performed in the same room for every participant. Voice quality and phonatory function were assessed by measuring changes in four objective acoustic measures and two clinician-based measures of voice perception. Phonatory tasks included repetition of ten sentences from the SDAI (Ludlow et al, 2018); these sentences have a high number of initial adductor phonemes, which tend to elicit symptoms in those with AdLD. Additional phonatory tasks included the production of the sustained vowel/ɑ/(as in “hot”) and other tasks required for the perceptual voice measures (described in more detail below). All voice and acoustic data analyses were completed by experienced investigators who were not present during data collection and who were blinded to group assignment (AdLD vs. Control) and time (pre- vs. post-rTMS).

We analyzed the voice recordings to derive four quantitative acoustic measures: jitter, shimmer, harmonics-to-noise ratio (HNR), and smoothed cepstral peak prominence (CPPS). Percent jitter, percent shimmer, and HNR (dB) are commonly used measures of voice quality that indicate the perturbation level of the signal wave (Carding et al. 2009). Jitter, shimmer, and HNR were obtained from one/ɑ/token per speaker before and after rTMS using Praat (Boersma and Weenink 2018). Jitter was measured as the average absolute difference between consecutive periods, divided by the average period, using the jitter (local) method. Shimmer was measured as the average absolute difference between the amplitudes of consecutive periods, divided by the average amplitude, using the shimmer (local) method. Harmonicity was measured as the mean HNR (Smiljanic and Gilbert 2017). Vowel productions for three participants with AdLD were excluded from the final analyses due to the presence of creak throughout most of the vowel duration at pre- and post-test, an issue likely related to AdLD symptoms. CPPS is an objective measure of general phonatory function, and it reflects the global relationship of periodic versus aperiodic energy in the voice acoustic signal (Patel et al. 2018; Watts et al. 2019). CPPS has been found to be correlated with perceived severity of voice symptoms and higher CPPS values represent a more normal vocal quality (Watts et al. 2019). CPPS (in decibels, dB) was extracted from acoustic recordings of the ten SDAI sentences using a custom script in Praat (Boersma and Weenink 2018). CPPS values were averaged across the ten sentences to derive an average CPPS for each participant before and after rTMS.

The number of phonatory breaks, a clinician-based measure frequently used in AdLD diagnosis and research (Ludlow 2008; Simonyan and Ludlow 2010, 2012), was analyzed. Participants repeated the ten sentences from the SDAI (Ludlow et al., 2018) before and after rTMS for identification of total phonatory breaks. A phonatory break is defined as an absence of voicing from a vocalic segment that is atypical to normally occurring phonetically related segments (i.e., voice onset time) (Sapienza et al. 2000). The total number of phonatory breaks was identified visually, by analysis of the acoustic waveform and auditorily (Sapienza et al. 2000), by a certified, licensed speech-language pathologist who specializes in voice disorders and who was blinded to group assignment and time. Phonatory breaks that occurred at word or syllable boundaries were not included in the analysis. Across all sentences analyzed in the AdLD group, 7% of recorded syllables were excluded from the count analysis due to extraneous noise in the acoustic signal. For each participant, the sum of the number of breaks in all ten sentences was compared before and after rTMS.

Another clinician-based measure used was the Consensus Auditory-Perceptual Evaluation of Voice (CAPE-V) (Kempster et al. 2009). The CAPE-V includes several 100-mm visual analog rating scales for assessment of voice quality in individuals with voice disorders and it has been used in the previous research on AdLD (Samargia et al. 2016; Simonyan and Ludlow 2010). It includes the repetition of six sentences, production of sustained vowels /ɑ/ (as in “hot”) and/i/ (as in “heat”), and a sample of spontaneous speech (approximately 20–60 s in duration). Six vocal attributes are evaluated with the CAPE-V and scores range from 0 to 100 with higher scores indicating a more impaired voice quality. The ratings for “Overall Severity” were of interest in this study, since it represents overall voice quality, and it is the most robust parameter of the CAPE-V in terms of inter-and intra-rater reliability (Carding et al. 2009); the other attributes were not reported due to the small sample size and pilot nature of this experiment. For each participant, the CAPE-V was scored by three raters: two licensed, certified speech-language pathologists, and one otolaryngologist who specializes in voice disorders. The assessors’ ratings were averaged for comparison of Overall Severity before and after rTMS.

### Data analysis

Safety and feasibility of the experimental procedures were analyzed with descriptive statistics.

The TMS and rTMS cSP threshold values were compared between groups using an Independent T test. Differences in cSP values after the single session of low-frequency rTMS were assessed with a repeated measures analysis of variance (RMANOVA) with Group (Control, AdLD) and TA side (left, right) as interaction factors against Time (Pre/Post). Given the age difference between groups (*t*_11_ = -2.692, *p* = 0.021), we ran the RMANOVA with age as a covariate.

All voice measures were averaged for each group at pre- and post-test. The effects of rTMS on all voice measures were explored with Cohen’s *d* effect sizes (with 95% confidence intervals) for changes between the pre- and post-test. Inter-rater reliability for the CAPE-V Overall Severity ratings was analyzed with the intra-class correlation (ICC) coefficient.

JMP (v15.1, SAS Co. Cary, USA) and R (R Core Team, 2013, Vienna, Austria) were used for statistical analyses. Significance level was *p* < 0.05 for all tests.

## Results

One Control participant (53 years old, female) did not complete the experiment because of EMG signal loss during the TMS pre-test. This participant was excluded from final analyses. The 13 remaining participants completed the full experiment and tolerated the testing and rTMS procedures. A total of 31 adverse events were reported at post-test and follow-up combined (Control = 16, AdLD = 15). The adverse events reported were expected and mild. Events reported included throat soreness (*n* = 10) and skin tenderness (*n* = 13) from the EMG electrode placement. Neck pain was reported by some participants (*n* = 5) which was likely related to discomfort associated with head positioning during TMS/rTMS procedures. Less common but expected adverse events reported included dental pain/discomfort (*n* = 1) and brief dizziness or fatigue (*n* = 2). Of note, no participants in the AdLD group reported worsening of voice symptoms after the experiment. A summary of all adverse events reported at each timepoint and per group is shown in Table 3.

**Fig. 1:**
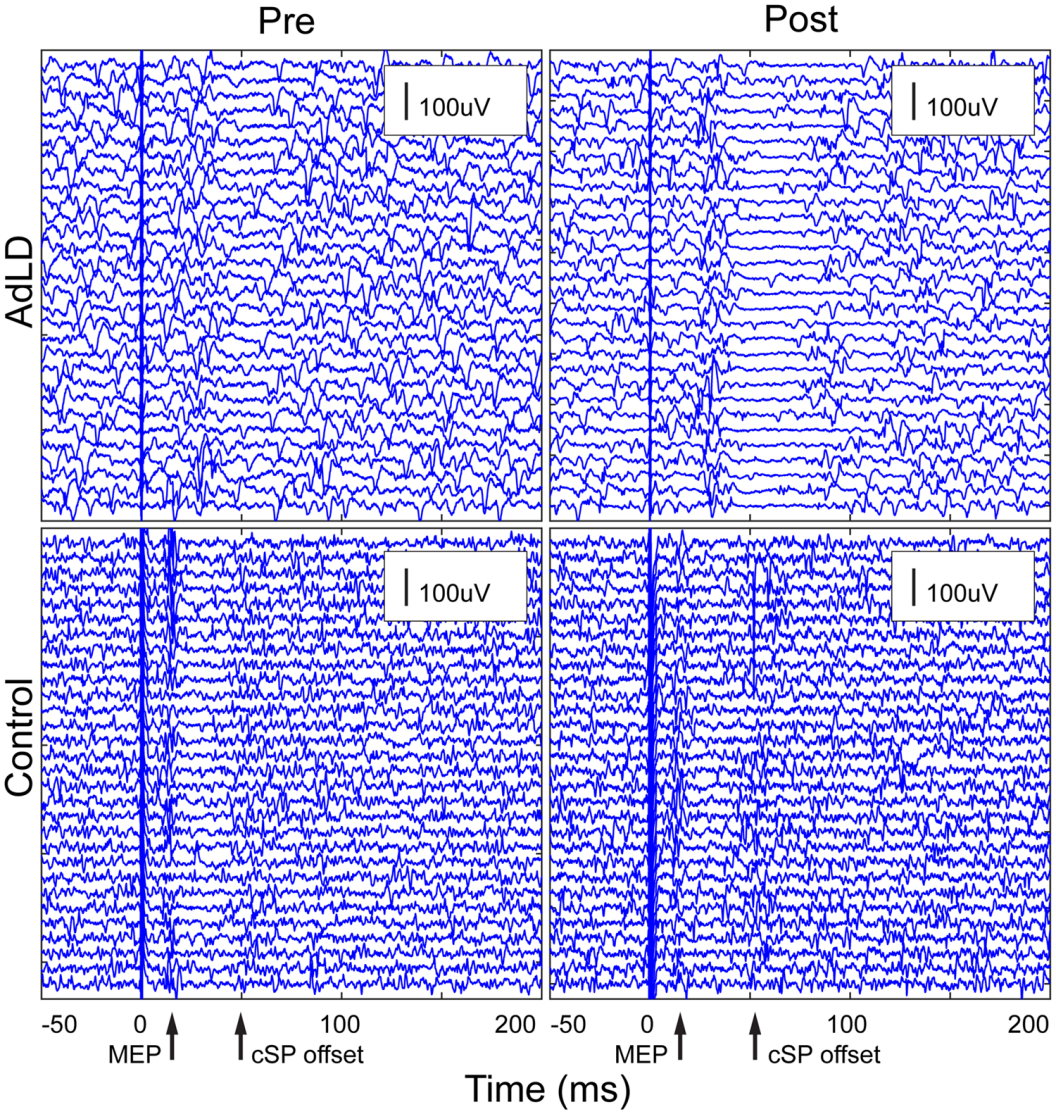
Representative raw EMG data of two participants demonstrating the cortical silent period (cSP) waterfall plot collected before (Pre) and after (Post) a single session of 1 Hz rTMS delivered to the laryngeal motor cortex. Each plot shows all 30 trials from one participant for each group before and after rTMS. *AdLD* adductor laryngeal dystonia, *EMG* electromyography, *MEP* motor-evoked potential

**Fig. 2:**
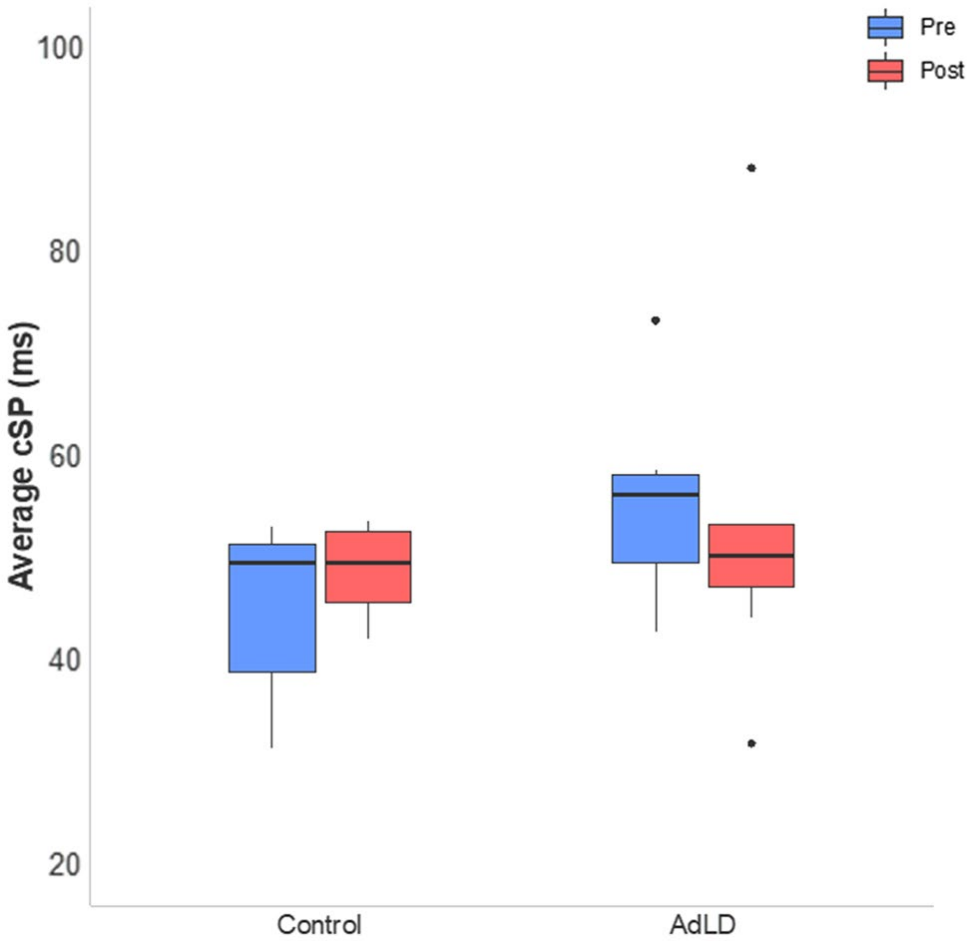
Average cortical silent period (cSP) duration for each group at pre- and post-test. There was no significant difference within or between groups. The values for left and right thyroarytenoid muscles were averaged, since there was no significant difference between sides (see text for details). *AdLD* adductor laryngeal dystonia; *ms* milliseconds

The average TMS and rTMS thresholds (% MSO) for eliciting a cSP are reported in Table 4. No significant differences between groups were found (TMS: *p* = 0.59; rTMS: *p* = 0.42). Figure 1 displays a representative sample a waterfall plot illustrating all 30 cSP trials for one participant from each group. cSP duration values for each group at pre- and post-rTMS are listed in Table 4. RMANOVA showed no significant effects of group (*F*_1,19_ = 0.013, *p* = 0.62), TA side (*F*_1,19_ = 0.001, *p* = 0.98), or age (*F*_1,19_ = 0.002, *p* = 0.84), and no interactions between factors (*F*_4,19_ = 0.06, *p* = 0.90). These results indicated no significant effects of the single session of 1 Hz frequency rTMS on cSP duration within or between groups. Given the lack of differences between TA sides within each group, we calculated a cSP average of the left and right TA for each participant at pre- and post-test prior to calculating effect sizes. In Controls, there was a medium (*d* = 0.50, CI [− 0.81, 1.80]) effect size in the hypothesized direction, meaning that there was prolonged cSP duration after rTMS. In the AdLD group, there was a negligible effect size indicating no change in cSP duration (*d* = − 0.17, CI [− 1.34, 1.00]). Figure 2 shows the average cSP duration for each group at pre- and post-test.

**Table 4:** Neurophysiology measures

ID	cSP thresholds (%MSO)		cSP, left TA (ms)		cSP, right TA (ms)		cSP CV (%)	
	TMS	rTMS	Pre	Post	Pre	Post	Pre	Post
Control	53.83 ± 7.8	55.83 ± 8.0	39.87 ± 7.3	45.22 ± 9.4	48.26 ± 13.5	54.07 ± 8.5	20.46	9.61
AdLD	56.71 ± 10.3	60.0 ± 9.5	54.04 ± 10.0	50.02 ± 18.3	56.74 ± 12.8	56.03 ± 17.0	17.62	32.47

Average (± SD) values for neurophysiology measures collected for all participants. There were no significant differences of cSP duration between pre- and post-rTMS

*AdLD* adductor laryngeal dystonia, *cSP* cortical silent period, *MSO* maximum stimulator output, *CV* coefficient of variation for the left and right TA combined, *TA* thyroarytenoid, *rTMS* repetitive transcranial magnetic stimulation, *TMS* single-pulse transcranial magnetic stimulation

**Fig. 3:**
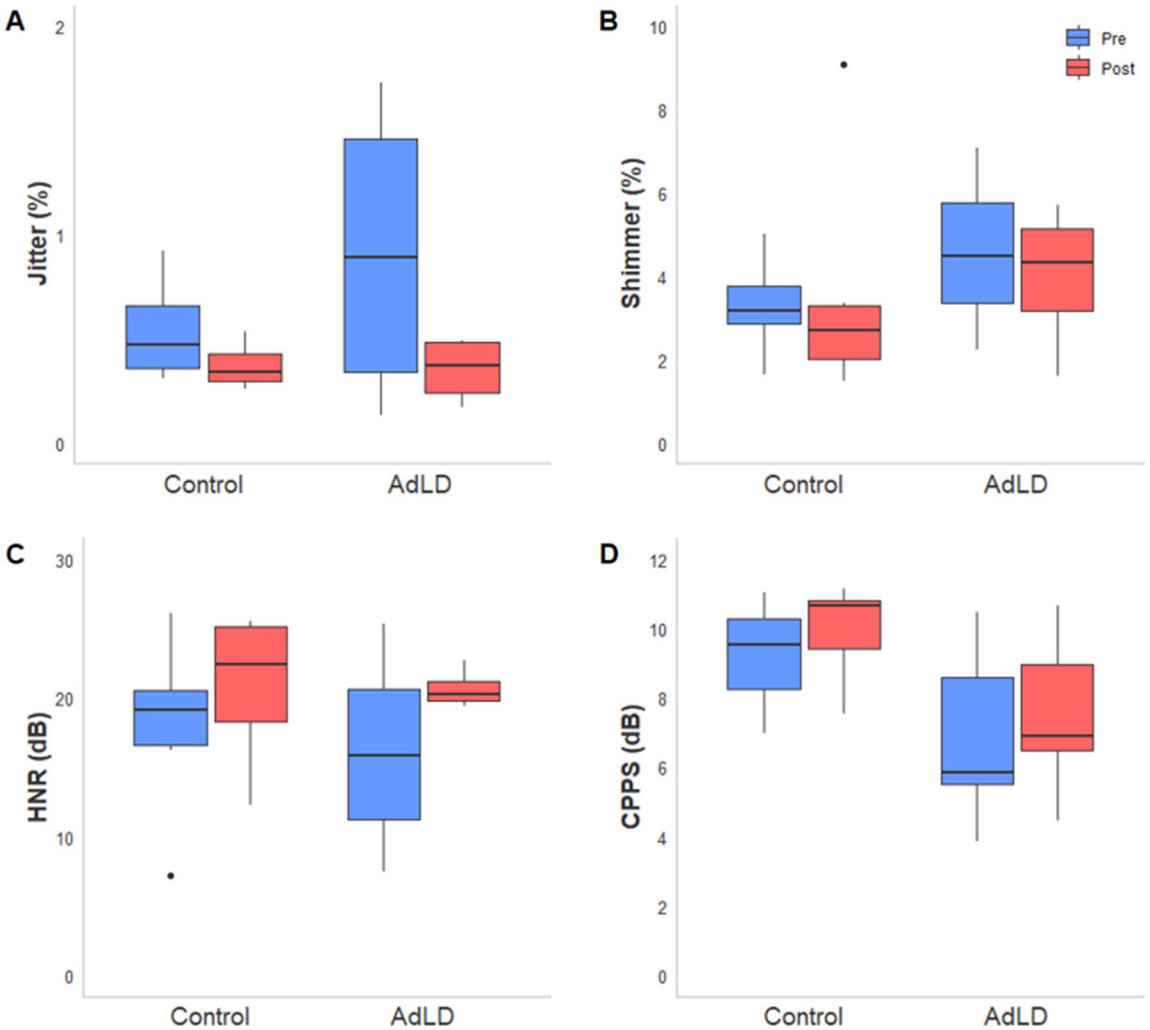
Objective measures of phonatory function for each group at pre- and post-test. **A**–**C** Jitter, shimmer, and harmonics-to-noise ratio during sustained phonation of the/ɑ/vowel. (D) CPPS results for the sentence “We eat eels everyday” of the Spasmodic Dysphonia Attribute Inventory. *AdLD* adductor laryngeal dystonia; *CPPS* smoothed cepstral peak prominence; *dB* decibels; *HNR* harmonics-to-noise ratio

**Fig. 4:**
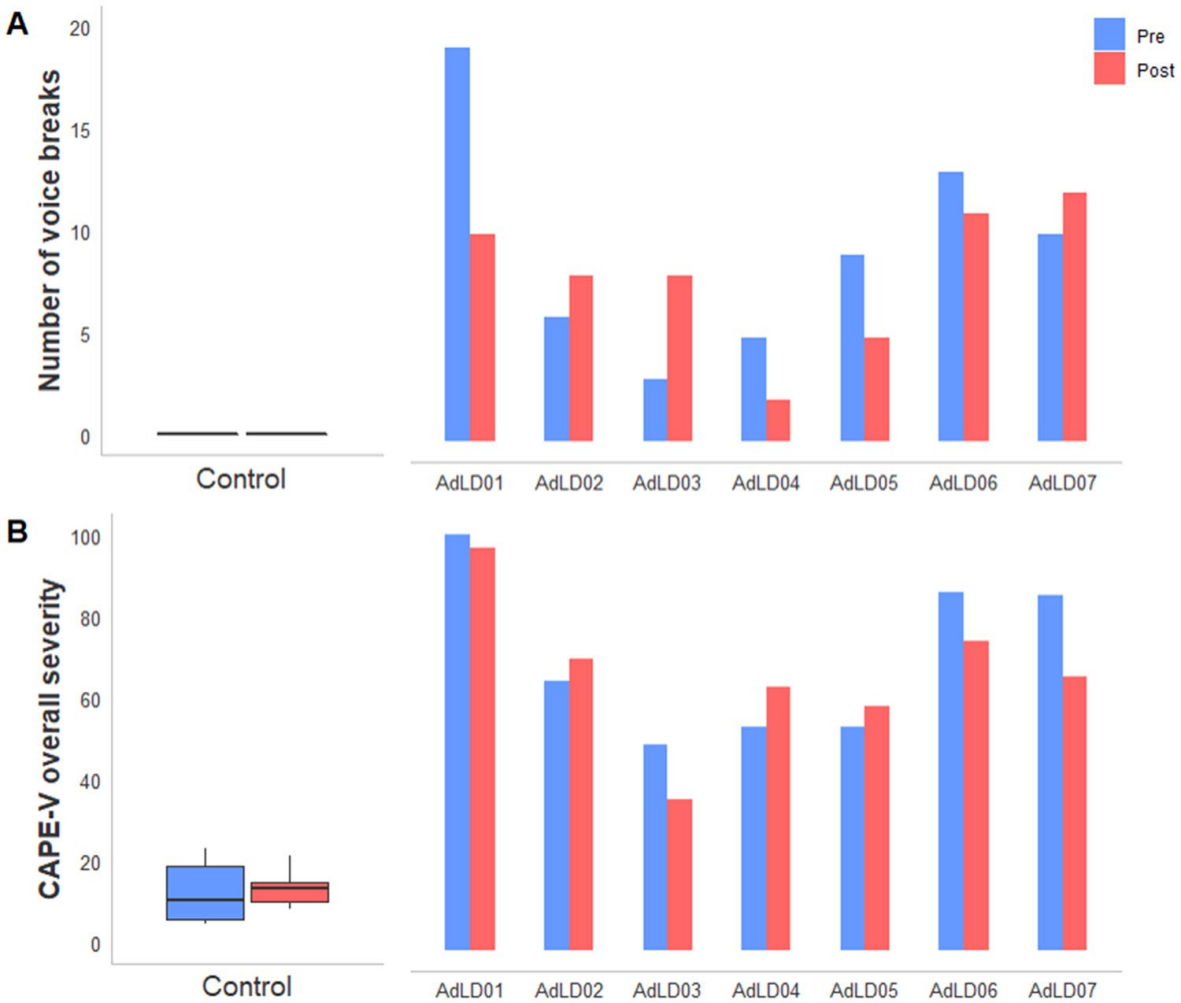
Pre- and post-rTMS changes in clinician-based voice measures. **A** Total phonatory breaks during the ten sentences of the Spasmodic Dysphonia Attribute Inventory; **B** Overall severity ratings on the CAPE-V. The Control group averages are shown for each measure along with the data for individual AdLD participants. *AdLD* adductor laryngeal dystonia; *CAPE-V* Consensus auditory-perceptual evaluation of voice

The voice assessment results are shown in Figs. 3 and 4. The acoustic measures of jitter, shimmer, and HNR demonstrated favorable change from pre- to post-rTMS in both groups (Fig. 3A–C). Jitter had large-effect sizes for the Control group (*d* = − 0.92, CI [− 2.27, 0.44]) and AdLD group (*d* = − 1.02, CI [− 2.90, 0.82]). Shimmer and HNR had small-effect sizes for the Control group (*d* = − 0.31, CI [− 1.60, 0.99] and *d* = 0.49, CI [− 0.81, 1.80], respectively), and large-effect sizes for the AdLD group (*d* = − 0.77, CI [− 2.56, 1.02] and *d* = 0.82, CI [− 0.98, 2.62], respectively). The CPPS for the average of all ten of the SDAI sentences did not reveal a change in the Control (*d* = 0.19, CI [-1.10, 1.48]) or the AdLD groups (*d* = 0.05, CI [− 1.11, 1.22]). Of all sentences analyzed, the acoustic CPPS from the sentence “We eat eels every day” indicated the most prominent change after rTMS with a small change in the direction of improvement in the AdLD group (*d* = 0.28, CI [− 0.89, 1.45]) and a medium effect in Controls (*d* = 0.50, CI [− 0.80, 1.81]) (Fig. 3D).

No phonatory breaks were detected in the Control group before or after rTMS, supporting a lack of voice disorder symptoms in the healthy participants. All participants in the AdLD group demonstrated phonatory breaks during symptomatic sentence production (Fig. 4A). Four out of the seven participants had a decrease in total number of phonatory breaks after rTMS, while three participants showed an increase in the same measure. On average, there was a small-effect size in the direction of decrease in phonatory breaks after rTMS for the AdLD group (*d* = − 0.28, CI [− 1.45, 0.89]).

The CAPE-V reliability between the three raters was excellent with an ICC coefficient of 0.96 for Overall Severity (CI [0.94, 0.98], *p* < 0.001). Four out of the seven participants had improved speech severity after rTMS. The CAPE-V Overall Severity had a small-effect size for the AdLD group from pre- to post-test (*d* = − 0.21, CI [− 1.37, 0.96]), indicating a small improvement in perceptual vocal quality after rTMS (Fig. 4B). There was no change in the CAPE-V Overall Severity within the healthy group from pre- to post-test (*d* = 0.14, CI [− 1.15, 1.43].

## Discussion

This pilot study assessed inhibitory laryngeal neuromodulation in healthy individuals and people with AdLD. rTMS was implemented using a novel technique (fine-wire electrode placement into the laryngeal muscles) to specifically target the TA cortical representation for the neuromodulation intervention. The study aimed to determine the safety and feasibility of the approach, as well as collect preliminary data on neurophysiologic and phonatory responses to rTMS intervention in a small group of participants. 1200 pulses of 1 Hz rTMS delivered to the LMC was safe and feasible. There was a large-effect size in the AdLD group after rTMS in acoustic measures of voice quality, and a small-effect size in decreased phonatory breaks and perceptual severity of symptoms. No significant effects on intracortical inhibition (cSP) were observed.

Thirteen out of the 14 participants enrolled completed the experiment. As reported in the Methods section, the EMG signal was lost in one participant due to movement of the fine-wire electrodes during TMS testing before the rTMS intervention. New electrodes could have been inserted, but time constraints on the data collection session led to the decision to terminate the experiment and exclude the participant from the final analysis. Overall, the results indicate that the TMS and rTMS procedures implemented in the current study are feasible.

All experimental procedures were well tolerated by participants. The distribution of adverse events reported was similar between groups and no unexpected events were reported. Throat soreness and tenderness around the insertion area were reported frequently in all groups and lasted up to 1 day after the experiment. These symptoms were expected and have been reported by other studies using fine-wire electrodes in intrinsic laryngeal muscles (Chen et al. 2017, 2020). Similarly, throat soreness and tenderness around the insertion area are often reported by patients with AdLD after botulinum toxin injections for treatment of the disorder. Reports of neck pain at post-test and follow-up suggest that head and neck positioning during TMS testing and rTMS delivery should be carefully considered. Importantly, there were no reports of exacerbation of symptoms in AdLD related to electrode insertion into the TA muscles, or to rTMS effects on the neurophysiology of the laryngeal musculature, indicating safety of application of 1 Hz rTMS to the LMC in AdLD.

Low-frequency rTMS has been previously explored as a possible intervention for focal dystonias with the goal of modulating cortical excitability of various brain regions. The rationale for using this neuromodulation modality is because when applied to contralateral corticomotor areas, low-frequency rTMS induces a decrease in cortical excitability that can persist beyond the stimulation period (Chen et al. 1997; Lefaucheur et al. 2014). Several studies tested the effects of 1 Hz rTMS over the contralateral primary motor or premotor cortices, or supplementary motor area in people with focal dystonias and reported no safety issues. Most studies focused on focal hand dystonia and found significant changes in cortical excitability, sometimes accompanied by improvement in movement performance and other symptoms (Borich et al. 2009; Kimberley et al. 2013, 2015; Lorezon et al. 2016; Obeso et al. 2016). However, there is yet no consensus for optimal parameters, stimulation site, or diagnostic indication for rTMS intervention in focal dystonias (Pirio-Richardson et al. 2017).

This study was not designed to measure efficacy of rTMS, but responses to neuromodulation were assessed. A 20-min session of low-frequency rTMS at sub-motor-threshold intensity was associated with large-effect sizes for improvement in voice perturbation measures (jitter, shimmer, and HNR) in the AdLD group, and trends in the direction of improvement for the number of phonatory breaks. These findings suggest beneficial effects in voice quality after a single session of rTMS. The pattern of improvement for the CPPS was most visible in the SDAI sentence containing all voiced phonemes which elicit glottal stops (“We eat eels everyday”). Production of the remaining sentences may not challenge the phonatory subsystem enough to be able to demonstrate such small changes in acoustic output. In a study of vibro-tactile stimulation in AdLD, 9 out of 13 subjects (69%) exhibited a reduction of voice breaks and/or a meaningful increase in CPPS, indicating improved voice quality (Khosravani et al. 2019). After low-frequency rTMS, 4 out of our 7 participants with AdLD (57%) had a decrease in total number of phonatory breaks after rTMS. Notably, 3 of the 4 subjects (AdLD01, 04, and 05) had the incidence of voice breaks nearly or more than halved which suggest that the methods employed in this study may be helpful for reducing voice breaks in a subset of individuals. These subjects also had an increase in CPPS after rTMS. It will be important for future work to examine characteristics associated with favorable response to rTMS.

It is important to note that even though jitter, shimmer, HNR, and CPPS are recommended as part of a comprehensive voice evaluation, these voice measures do not specifically assess the clinical symptoms of AdLD and, therefore, the changes observed in this study may not necessarily have impact on the unique impairments experienced by patients with AdLD. However, these voice perturbation measures have been previously shown to be sensitive to changes in voice quality in a number of patient populations (Carding et al. 2009), which provides confidence that the changes observed in the current study signal an improvement in acoustically derived vocal quality. In addition, the potential effects of repeating phonatory tasks within one session are unknown. It is possible that differences in phonatory production of the same tasks before and after the rTMS session was due to familiarization to the stimuli. This may be the reason that the Control group showed changes after rTMS in jitter, shimmer, HNR, and CPPS. Overcoming this potential limitation would have required additional sessions or changing tasks from pre to post, which would introduce another set of potential confounds. Future investigations should consider including a larger number of measures such as cepstral spectral index of dysphonia (CSID) and aerodynamics measures to help detect any potential phonatory changes in response to 1 Hz rTMS.

The CAPE-V Overall Severity, a perceptual measure of voice quality, had only a small-effect size in the direction of improvement after one session of rTMS in the participants with AdLD. This was not unexpected, since a single session of neuromodulation is unlikely to induce significant perceptual changes in voice quality. Perceptual measures of voice impairment are well known to be subject to bias and other listener factors that may decrease their sensitivity to small changes in voice production in comparison to acoustic measures. In fact, the previous studies examining the effect of a variety of interventions on voice quality found that improvements in more objective acoustic measures, and even patient-reported outcomes, outperformed improvements in auditory-perceptual measures (Kapsner-Smith et al. 2015; Speyer et al. 2004; Watts et al. 2019).

We did not detect significant neurophysiologic (cSP) change following the single session of 1 Hz rTMS in either group. Multiple factors may have contributed to the lack of significant effect on cortical inhibition, such as the small sample size, short duration of stimulation, variability in cSP response, or suboptimal intensity of the rTMS intervention. Perhaps, multiple and/or longer sessions are needed for measurable changes to be detected, as suggested by rTMS studies in other dystonias (Borich et al. 2009; Kimberley et al. 2013, 2015; Lorezon et al. 2016; Obeso et al. 2016). In addition, cSP responses were quantified immediately after rTMS and, thus, any delayed neuromodulatory effects were not measured. It is possible that the lack of change in cSP was related to the TA activation level during rTMS, since this muscle is always active during breathing and some studies indicate that better responses to 1 Hz rTMS are observed when the muscle is at rest (Klomjai et al. 2015). Because it is not possible to apply neuromodulation with the TA completely at rest, future investigations may need to explore different stimulation parameters than the ones tested in the current study. It is also conceivable that beneficial clinical effects do not require sustained changes in cortical excitability, suggesting that a link between the neurophysiologic and clinical measures is not required. Indeed, studies of low-frequency rTMS in other disorders, such as dysphagia, aphasia, and bruxism, reported clinical benefits with no or mixed neurophysiological responses (Lefaucheur et al. 2014; Michou et al. 2016; Zhou et al. 2016).

### Limitations and challenges

This pilot investigation had some limitations that future work may address. The principal limitation was the lack of a sham condition for comparison with active rTMS to allow testing for placebo effects of the intervention. A sham condition was not included, because it would require either a larger sample or crossover sessions for each group which would have made recruitment even more challenging for an experiment that was already time intensive. Furthermore, given that there are virtually no known treatments that induce immediate changes in phonatory function in this disorder, the risk of a false positive effect was low. Additionally, all assessments were completed off-line by investigators blinded to group or testing session (pre/post).

This study involved only a single session of inhibitory rTMS. To establish clinically meaningful changes, a clinical trial will likely require many days or weeks of treatment. It was not expected that a single dose would produce significant perceptual voice changes. In all diagnoses that have shown efficacy of rTMS, multiple sessions of treatment are required (Lefaucheur et al. 2014; Lorezon et al. 2016; Obeso et al. 2016). However, the undertaking of a large-scale trial with multiple days of treatment requires prior signal of potential success with a given set of parameters as well as evidence that the treatment does not exacerbate the condition. Therefore, a preliminary study like the current investigation was necessary to establish safety, feasibility, and effects of a single rTMS session given the novel methods and clinical population to indicate whether a larger trial with multiple sessions is warranted.

The cSP was the only neurophysiologic outcome used which limited the ability to measure other possible effects of 1 Hz rTMS. This methodological limitation was due to the inherent tonic activity in the TA muscle during breathing which does not allow measurement of other TMS outcomes, such as the motor-evoked potential, or use of paired pulse protocols, because these techniques require a resting muscle (Chen et al. 2020).

We acknowledge the variability in the injection schedule of the AdLD group. Considering the known variability in the duration of botulinum toxin effects in patients with AdLD and other dystonias, future work could include an additional group of untreated patients and/or multiple rTMS sessions within-subjects to investigate rTMS efficacy during different stages of botulinum toxin effects.

Finally, there was lack of age and sex match between the Control and AdLD groups; the Control group was younger (Control vs. AdLD, *p* = 0.021) and included more males. These dissimilarities were due to the higher percentage of females with AdLD diagnosis and the challenge of recruiting healthy older individuals willing to undergo the experimental procedures involved in the study. This potential confound was minimized by adjusting the RMANOVA for age.

## Conclusions

This pilot study explored the effects of rTMS targeting the LMC and the affected musculature in AdLD. Given that rTMS has not been previously tested in this clinical population and the novel technique involving fine-wire insertion into the TA, initial safety and feasibility testing in a small group of people with AdLD were warranted. One session of low-frequency rTMS to the LMC was a safe and feasible intervention that demonstrated trends of beneficial effects on voice quality and phonatory function in AdLD. The promising, albeit subperceptual improvements in vocal quality and phonatory function found in the current study offer support for future work to test potential clinical benefits of multiple sessions of 1 Hz rTMS in a larger patient sample.

Although it is early for clinical implementation of rTMS in AdLD, it is important to consider the methods that could be used for upcoming randomized controlled trials and future clinical feasibility. Establishing clinically meaningful changes will likely require multiple rTMS sessions across many days or weeks of treatment. For optimal, individualized targeting of rTMS in AdLD, single-pulse TMS with fine-wire EMG to laryngeal muscles may be required, as implemented in this study. This invasive technique is required to assess laryngeal-specific cortical excitability at baseline and post-intervention effects. However, measuring TA EMG activity will likely not be required during repeated sessions of rTMS, as use of the neuronavigation system to track the rTMS coil should be sufficient to ensure consistent delivery of rTMS across multiple intervention days. Eliminating the need for fine-wire TA EMG on intervention days should help increase future clinical feasibility and future work can explore the effects of individualized vs. targeted neuromodulation delivery.

## Supplementary Information

Below is the link to the electronic supplementary material.

Supplementary file1 (DOC 37 kb)

## Data Availability

The datasets generated during and/or analyzed during the current study are available from the corresponding author on reasonable request.
