# Supplementary material for: Effects of low-frequency repetitive transcranial magnetic stimulation in adductor laryngeal dystonia: a safety, feasibility, and pilot study
Source: Exp Brain Res. 2021 Dec 2;240(2):561–74. doi: 10.1007/s00221-021-06277-4 (PMC8858301; doi:10.1007/s00221-021-06277-4)
Supplement: Supplementary file 1 — Supplementary file1 (DOC 37 kb) [file 221_2021_6277_MOESM1_ESM.doc]

**SUPPLEMENTAL MATERIAL**

**Supplement 1: Subject report of symptoms**

Subject ID: _____________ Date of experiment: _____________

| **Symptom** | **Pre-test** | **Post-test** | **Follow-up** |
| --- | --- | --- | --- |
| Seizure |  |  |  |
| Headache* |  |  |  |
| Throat pain (sore throat)* |  |  |  |
| Tenderness on skin (insertion area)* |  |  |  |
| Neck pain* |  |  |  |
| Dental pain* |  |  |  |
| Hearing discomfort* |  |  |  |
| Nausea |  |  |  |
| Dizziness |  |  |  |
| Abnormal sleep |  |  |  |
| Difficulty with concentration |  |  |  |
| Anxiety* |  |  |  |
| Memory issues |  |  |  |
| Mood change |  |  |  |
| Balance |  |  |  |
| Other (describe) |  |  |  |

* If present, rate from 1-10
